# Free-breathing 2D Cine DENSE with Localized Excitation, Self-navigation and Motion Correction

**DOI:** 10.1186/1532-429X-18-S1-P319

**Published:** 2016-01-27

**Authors:** Xiaoying Cai, Xiao Chen, Yang Yang, Michael Salerno, Daniel Weller, Craig H Meyer, Frederick H Epstein

**Affiliations:** 1 grid.27755.32000000009136933XBiomedical Engineering, University of Virginia, Charlottesville, VA USA; 2 Siemens Medical Solutions, Princeton, NJ USA; 3 grid.27755.32000000009136933XElectrical and Computer Engineering, University of Virginia, Charlottesville, VA USA; 4 grid.27755.32000000009136933XRadiology, University of Virginia, Charlottesville, VA USA; 5 grid.27755.32000000009136933XCardiology, University of Virginia, Charlottesville, VA USA

## Background

Cine DENSE MRI is an established myocardial strain imaging technique with high accuracy and rapid data analysis. Current cine DENSE protocols utilize breath-holding (BH); however, this practice limits the use of this technique to patients with good BH capabilities, excluding many pediatric and some heart failure patients. We propose a free-breathing (FB) framework for cine DENSE that uses localized excitation, self-navigated motion estimation, and k-space correction to enable FB motion-corrected (MC) imaging.

## Methods

**Figure 1:**
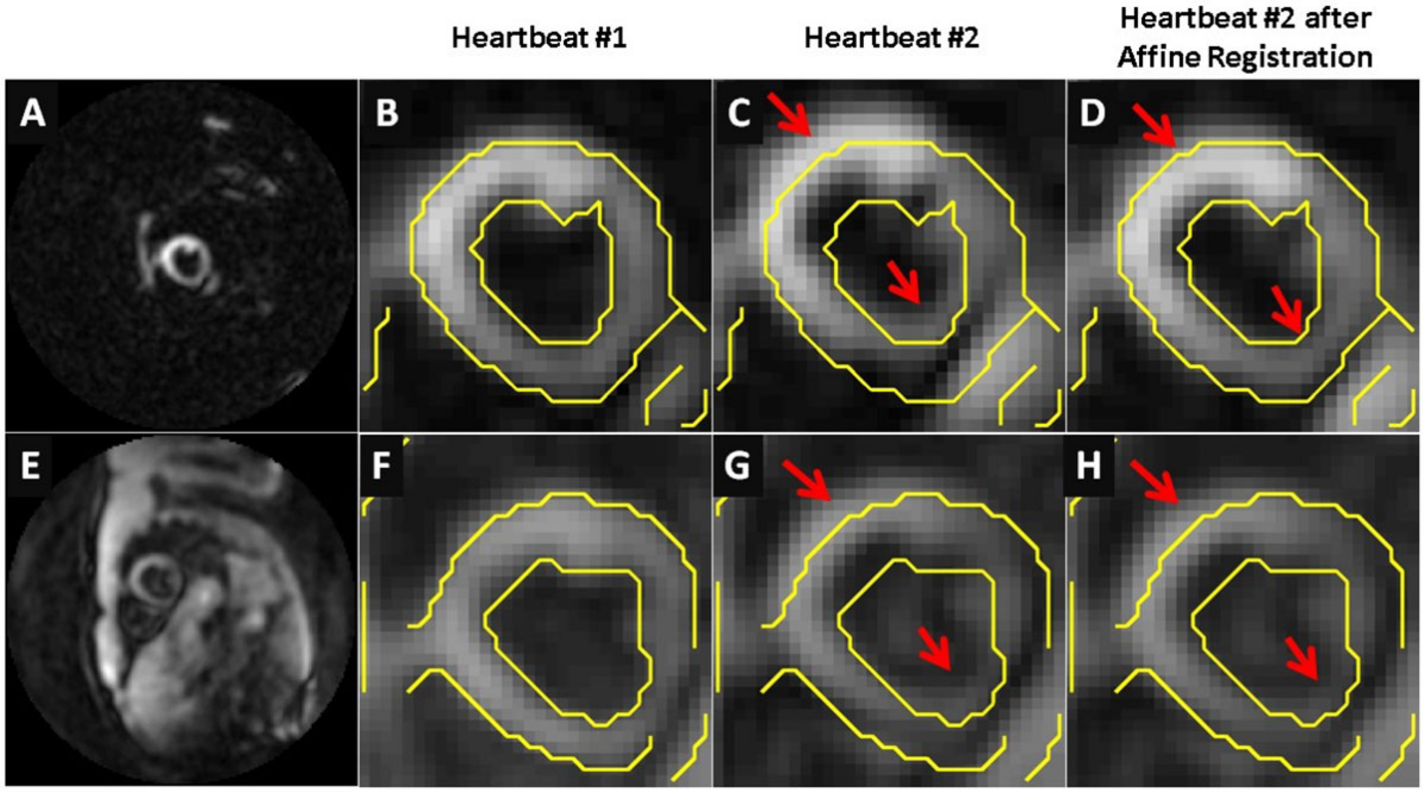
**Example sliding-window intermediate images used for estimation of inter-heartbeat cardiac motion due to breathing from scans with (A-D) and without (E-H) localized excitation**. Heartbeat #2 (C,G) was registered to heartbeat #1(B,F), as shown in (D,H). Localized excitation facilitated more accurate estimation of the effects of breathing on cardiac motion.

A modified cine DENSE sequence was implemented on a 1.5T scanner (Avanto, Siemens). The sequence utilized an ECG-gated segmented acquisition with a variable-density spiral trajectory, typically requiring 14 heartbeats to acquire a full 2D cine dataset encoded for 2D in-plane displacement. Localized excitation was implemented by applying orthogonal slice-selective RF pulses in the DENSE preparation so that displacement-encoded stimulated echoes would only occur in the heart region where the slice profiles intersect. The use of localized excitation facilitated automatic estimation of heart motion due to respiration, as other tissues such as liver and chest did not generate significant signal (Figure 1, A,E). Low-resolution intermediate images were reconstructed each heartbeat by an intra-heartbeat sliding-window method and image registration was used to estimate inter-heartbeat respiratory motion. Affine motion correction was applied in k-space, followed by final reconstruction of high-resolution cine DENSE images. Mid-ventricular short-axis 2D cine DENSE datasets were collected from 7 healthy volunteers using a 6-channel coil, temporal resolution = 28 ms, in-plane resolution = 2.3 × 2.3 mm^2^, slice thickness = 8 mm, localized excitation width = 80 mm, field of view = 160 mm, number of spiral interleaves per image = 4, and number of interleaves acquired per heartbeat = 2. Images were reconstructed offline with and without MC. Displacement and strain analysis was performed for 36 segments. To assess for variability due to respiratory motion, the coefficient of variation (CV) of end-systolic strain was compared among the three groups: BH, FB with and without MC using one-way ANOVA.

## Results

**Figure 2:**
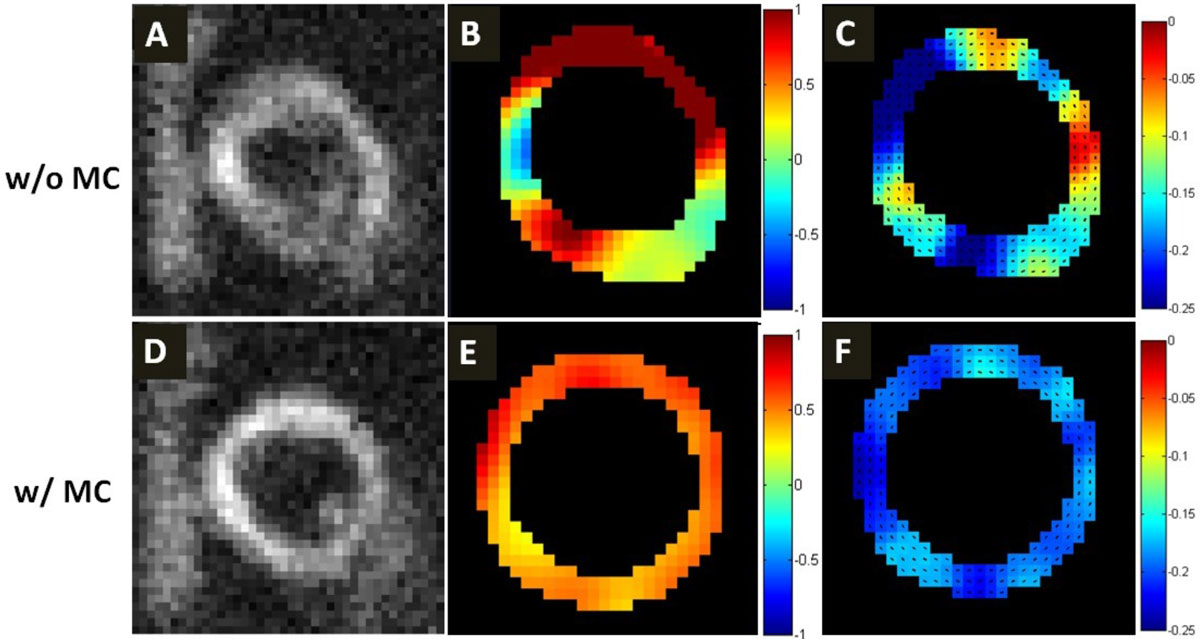
**Example magnitude-reconstructed images (A,D) and corresponding displacement maps (B,E) and strain maps (C,F) reconstructed without (top) and with (bottom) MC**.

Figure 1 demonstrates that affine registration estimated respiratory motion of the heart more accurately in the images acquired with localized excitation as compared to those obtained with a full-FOV acquisition. Figure 2 demonstrates that magnitude images reconstructed with MC presented less blurring compared to without MC, and that displacement and circumferential strain maps obtained with MC showed less variability. The CV (mean standard deviation) of end-systolic strain in 7 volunteers was similar for BH and FB with MC (0.12 ± 0.05, 0.13 ± 0.09, respectively, p = 0.97), but much higher for FB without MC (0.27 ± 0.17, p < 0.05 vs BH and FB with MC).

## Conclusions

FB cine DENSE shows good potential for strain imaging in patients with sub-optimal BH capabilities.

